# Particular promotion of the intrauterine system to “vulnerable” adolescents: a qualitative exploration of hormonal contraceptive prescription practices

**DOI:** 10.1186/s12978-026-02388-9

**Published:** 2026-06-17

**Authors:** Marita Kallesten Brønnick, Jone Ravndal Bjørnestad, Daniel Adrian Lungu, Tore Tjora

**Affiliations:** 1 Department of Psychology, Faculty of Social Sciences, University of Stavanger, Stavanger, Norway; 2 TIPS – Centre for Clinical Research in Psychosis, Stavanger University Hospital, Stavanger, Norway; 3 Department of Psychiatry, District General Hospital of Førde, Førde, Norway; 4 SHARE – Centre for Resilience in Healthcare, Faculty of Health Sciences, University of Stavanger, Stavanger, Norway

**Keywords:** Adolescents, Intrauterine system, Hormonal contraceptives, Prescription, Depression, Mental health

## Abstract

**Background:**

Previous studies have suggested that the intrauterine system increases the risk of depression, particularly in adolescents. However, these studies relied on national registry data collected at a time when medical guidelines discouraged adolescent use of this contraceptive method. As a result, the adolescents who appeared in these registries represented an atypical group who had this contraceptive prescribed against medical recommendations. Consequently, unknown or unmeasured participant characteristics may have introduced confounding variables and influenced conclusions. Although prescription guidelines for intrauterine systems now include adolescents, selective prescribing may persist. If so, it could affect future studies on hormonal contraceptives, even though the decision is clinically sound. We thus aimed to explore current contraceptive prescription practices, investigating user characteristics, policies, and contextual factors that might influence prescribing the intrauterine system rather than other hormonal contraceptive products, or vice versa.

**Methods:**

We conducted focus group interviews with 23 healthcare professionals employed at six health centers in Norway, prescribing hormonal contraceptive drugs to adolescent women. We used thematic analysis to identify factors that can influence which products are recommended and requested.

**Results:**

Our analysis generated three themes: “Old conceptions die hard” describes skepticism among family doctors and mothers regarding adolescent use of the intrauterine system, “Particular promotion of the intrauterine system to “vulnerable” adolescents” outlines the practice of healthcare professionals recommending this contraceptive method to individuals identified as vulnerable, and “What tips the scale: Pros, peeves and other people” discusses influences on adolescents’ contraceptive choice.

**Conclusions:**

This exploratory study suggests that both prescription practices and personal preferences influence choice of contraceptive product, and that it is not arbitrary who has the intrauterine device inserted. Future quantitative research should incorporate the factors identified in this exploration, to minimize their confounding effects when aiming to draw causal conclusions on the effects of the intrauterine system on mental health.

**Supplementary Information:**

The online version contains supplementary material available at https://doi.org/10.1186/s12978-026-02388-9.

## Abstract (plain language summary)

The intrauterine system is an effective, easy-to-use hormonal contraceptive method. However, some have raised concerns that it can cause symptoms of depression in adolescents. We believe these concerns to be misguided, as they stem from studies on teenage users conducted at a time when medical guidelines advised against this contraceptive method in adolescents. Consequently, the teenagers included in these studies may already have had medical or mental health conditions that led clinicians to prescribe the intrauterine system despite the guidelines. These underlying conditions may also explain the later development of depressive symptoms, which may then have been incorrectly attributed to using the intrauterine system. Recommendations for the intrauterine system now include adolescent women, but if different contraceptives are still selectively prescribed, the conclusions we draw when researching these drugs and mental health could be affected.

We therefore explored current prescription practices, interviewing healthcare professionals at health centers for adolescents. We aimed to identify factors that could influence which type of contraception adolescent women prefer and what healthcare professionals prefer to prescribe.

Our informants said that several factors can still influence who has the intrauterine system prescribed. Most notably, specific individual characteristics were described as leading to some adolescents being viewed as “vulnerable”. For these adolescents the healthcare professionals reported making additional efforts to recommend the intrauterine system over other types of contraception, because of its effectiveness and user friendliness.

This study highlights the need for assessing background factors when researching the relationship between hormonal contraceptives and mental health.

**Supplementary Information:**

The online version contains supplementary material available at https://doi.org/10.1186/s12978-026-02388-9.

## Introduction

The intrauterine system (IUS) is the most frequently used hormonal contraceptive (HC) worldwide [1]. It requires minimal attention once inserted and is among the most effective methods available for preventing pregnancy [2]. While previously used primarily by adult women, the IUS is now gaining increasing popularity also in the adolescent population [3, 4]. As teenage pregnancy is associated with several adverse social- and health-related outcomes [5], safe and efficient contraception is especially important in this group. In addition to contraceptive efficacy, the IUS can confer non-contraceptive benefits such as reduced menstrual bleeding and pain, ameliorated symptoms of endometriosis, and decreased risk of endometrial and ovarian cancers [6–8]. Although these benefits are well established, some posit that the IUS can have harmful effects on mental health, especially when used by adolescent women. This notion arose upon the publication of a Danish, nationwide cohort study by Skovlund et al. [9], which suggested that adolescent IUS use is associated with a significantly increased risk of depression and use of antidepressant drugs. The authors found the risk to be increased when compared with both non-users and users of all other HC products. This paper has had extensive impact, being cited both in popular science communication and in academic publications, and it has been referred to as representing a “shift” in the research on HCs and depression [10, 11]. However, this study was based on information from national registries, and these types of studies can suffer from confounding introduced by variables that are unknown or unaccounted for. The Skovlund study may have been influenced by a selection bias, as the authors analyzed data registered from 1995 to 2013. This timing is important, because while the IUS is currently recommended for adult and adolescent women alike; teenagers using the IUS in the 1990s and early 2000s did so against the medical guidelines of that time. Consequently, these adolescents may have exhibited characteristics that could be causally linked to both the later occurrence of depression and to the prescribers’ decision to place an IUS against medical recommendations.

The considerations leading to restricted recommendations included concerns about pain and difficulties when inserting the device, as nulliparous women have a narrow uterine cervix [12]. Moreover, reports on a previously available, non-hormone-releasing intrauterine device that was retracted from the market in 1974 [13] gave rise to widespread beliefs that all intrauterine contraception could cause pelvic inflammations and subsequent infertility [14]. Although later disproven, this perception persisted and contributed to reluctance in recommending the IUS to adolescent women. As such, when the levonorgestrel-releasing intrauterine system was released in Europe in the 1990s, and in the United States in 2000, it was approved for and marketed towards adult women who had previously given birth. In countries like Norway and Denmark, the guidelines for this IUS said “*not the method of choice”* for young, nulliparous women (Danish Medicines Agency, personal communication, December 2024, Norwegian Medical Products Agency, personal communication, January 2025) and the United States drug labeling said “*recommended for women who have had at least one child*” [15]. Consequently, adolescent use was uncommon in the years that followed, and it wasn’t until 2013 that a new, low-dose IUS was marketed specifically towards this group [16]. Currently, the levonorgestrel-releasing IUS is available in three different dosages and adolescent women are included in recommendations for all three [17].

This shift in recommendations on adolescent IUS use could explain results from more recent registry studies that analyzed data collected in Sweden from 2010 to 2017 [18, 19] and found a weaker association between adolescent IUS use and depression than the one indicated by Skovlund et al. [9]. The latter study noted that the association diminished even more towards the end of the study period, once adolescent IUS use became more common [19]. In line with our reasoning, the authors also stated that despite these observations, residual confounding—arising from factors that influence both prescription of the IUS and the risk for depression—may still be present and lead to an overestimation of risk. To our knowledge, all findings on the IUS and adolescent mental health are derived from registry studies with limited possibilities for sorting out potential confounders.

Given the uncertainty associated with these findings, along with the shifting trends in IUS use, we believe there is a need to take a step back and explore the current landscape of adolescent hormonal contraceptive use. We consider a qualitative exploration of prescription practices a good place to start, as those prescribing these products encounter a broad range of individuals seeking contraceptive advice. Prescribers may therefore convey insight and share experiences regarding adolescents’ preferences. Moreover, the prescribers may themselves act as gatekeepers that influence adolescents’ choice. An open exploration of prescription practices could thus reveal systemic, prescriber- and user-associated factors potentially confounding registry- and other observational or quasi-experimental studies. The factors identified in this exploration may inform the design of future quantitative studies on the IUS and mental health, thereby reducing confounding. Our research question is thus: Which user characteristics, user preferences, healthcare professional practices, policy or other structural aspects might influence prescription of the IUS?

## Materials and methods

The study is reported in accordance with the Consolidated Criteria for Reporting Qualitative Research (COREQ) checklist (See supplementary materials).

### Scientific framework

Our project was carried out within a framework of critical realism. Critical realism aims to contribute knowledge about reality, acknowledging the complex social dimensions that interact with this reality [20]. We aimed to explore current guidelines and principles for prescription, along with user- and healthcare professional inclinations that might influence the ways in which these are understood and applied. Rather than taking a phenomenological deep dive into participants’ lived experiences, we used this explorative, inductive design to identify *possible* influences on prescription practices, with the aim of informing future, quantitative studies.

Reflexivity was attended to by engaging in ongoing dialogues within the research team, recognizing that researchers’ pre-understanding, experiences, and perspectives are not independent of analytical processes. The research team consisted of professionals from complementary disciplinary backgrounds, namely foundational medical studies, nursing, sexology and applied neuroscience (MKB), psychology (TT and JRB), and health services research (DAL). This breadth of perspective informed the analytical process, allowing us to approach the data from clinical, psychological, and organizational vantage points. While we acknowledge that our familiarity with reproductive health, psychology, and health service research may have sensitized us to certain themes while potentially obscuring others, our multidisciplinary background served as a partial counterweight to this risk, as divergent professional perspectives were brought to the table during collaborative analytical discussions. Prior to data collection, we held a shared assumption that individual characteristics of adolescent women might influence healthcare provider recommendations regarding hormonal contraceptives. However, we did not enter the process with expectations about which characteristics would prove salient. This open stance supported an inductive orientation during thematic analysis and helped guard against premature conclusions. The choice of critical realism as framework further supported reflexive practice, enabling us to treat participants’ accounts as meaningful data about real-world mechanisms without assuming a single disciplinary lens through which to interpret them.

### Recruitment

We contacted all the largest regional health centers for adolescents in Rogaland, Norway, and several smaller ones—collectively covering the major parts of the county. These centers were purposively selected because they are the primary public health service providers offering contraceptive counselling and prescription to adolescents in Norway, and they are staffed by all Norwegian healthcare professionals authorized to prescribe these substances (public health nurses, midwives, gynecologists and general practitioners) [21]. We requested at least two healthcare professionals from each center, who should be authorized hormonal contraceptive prescribers with at least one year of experience working with adolescents. All centers conveyed an interest in participating, but some could not provide enough participants of the required background and/or experience. Two set up an initial appointment but had to cancel due to illness among staff.

### Participant characteristics

We conducted six focus group interviews, one at each of six health centers for adolescents, with a total of 23 participants. Group sizes ranged from two to six. Only participants and researchers were present. Our sample (*n* = 23) consisted entirely of female employees of health centers for adolescents in the southwestern part of Norway. Nineteen were public health nurses, two were midwives, and two were general medical practitioners. Age range was 38–63, mean age was 52, and years of experience prescribing HCs to young women ranged from 1.5 to 22, with a mean of 10. Health centers for adolescents are staffed mainly by public health nurses and medical practitioners, with the former being represented in higher numbers. Some centers also employ midwives [21]. These centers are the main providers of contraceptive counselling and prescribing in Norway, and participant composition thus reflects the structure of Norwegian adolescent health services.

### Interviews

Interviews were conducted between June and December 2024 and lasted about 90 minutes each. Our interview guide (see supplementary materials) was developed with the aim of exploring (1) Choice of product: who do adolescent women listen to, and what are healthcare professionals’ guidelines and practices, (2) Long-acting reversible contraceptives such as the IUS and the subdermal implant: goals, preferences and concerns, from both adolescent and healthcare professional perspectives, (3) HC side-effects, and (4) Trends in prescription practices. We asked open-ended questions, encouraging detailed descriptions of participants’ perspectives and practices, prompting elaboration on specific subtopics if the conversation strayed from our research question. We piloted our guide with a public health nurse previously employed at a health center for adolescents, currently holding an academic position at our university, and adjusted our guide in accordance with her input. The first three interviews were carried out by researchers MKB (female, foundational medical studies, nurse, master’s in sexology, master’s in applied neuroscience, PhD candidate) and TT (male, clinical psychologist, associate professor), and the last three interviews were conducted by MKB alone. Participants were informed about interviewer occupation and affiliation, as well as association with a PhD project. We met with participants and collected data until we deemed that we had reached sufficient information power, in the sense used by Malterud et al. [22]. This was assessed iteratively across the data collection process rather than retrospectively. Following focus group interviews, the research team reviewed emerging themes and considered whether new analytical ground was being covered. By the final interviews, we observed that participants were raising perspectives and concerns that had already been brought forth in earlier groups, with no substantially new themes emerging. This pattern gave us confidence that the dataset had reached a level of thematic sufficiency adequate for addressing the research question, which was relatively narrow in aim. Additionally, participants shared a specific professional role, while still exhibiting variation in their experiences, contributing to dense sample specificity. Moreover, the dialogue was strong, and the data were rich in relevant detail. These features support the credibility of the information power judgment despite a moderate total sample size [22].

### Ethical considerations and data management

In line with the Declaration of Helsinki and European ethical standards [23], consent forms and information sheets were signed by participants prior to data being collected. All participants were thoroughly informed about the project’s purpose, benefits, risks, and what was required from them, as well as data management and participants’ right to withdraw their consent without any consequences for them. Interviews were recorded, encrypted and stored safely within the University of Oslo’s Nettskjema-dictaphone app [24], accessible only to authors MKB and TT via two-factor authentication login. The project was approved by the Norwegian Agency for Shared Services in Education and Research (Sikt) and deemed to be in accordance with data protection regulations (reference #375555, 7th of June 2024).

### Analysis

We conducted thematic analysis in accordance with the principles outlined by Braun and Clarke [25]. NVivo 15 was used to organize and manage data [26]. We searched for meaning across the entire data corpus but only extracted data relevant to our research question. Themes were identified inductively, using a six-step approach: (1) Authors MKB and TT independently familiarized themselves with the data, and (2) generated initial codes based on a semantic condensing process. (3) These codes were then grouped into potential themes. (4) MKB and TT compared and reviewed the themes, reaching agreement on five themes considered pertinent to our research question, and then generated a thematic map of the analysis. (5) This map was subsequently presented to the rest of the team in a collaborative meeting. The themes were discussed and named, and the five initial themes were condensed into three final themes that all authors agreed captured essential elements of prescription practices, as well as factors influencing choice of HC. Participant quotes were translated from Norwegian to English by MKB, and the translation was then validated and approved by all other authors. (6) All authors contributed to the production of the final report [25].

## Results

Three main themes pertaining to our research question were established. These concerned ideas of the past affecting practices of today, differential prescribing of different HCs, and influences on adolescents’ choice. We named them “Old conceptions die hard”, “Particular promotion of the intrauterine system to “vulnerable” adolescents”, and “What tips the scale: Pros, peeves and other people”.

### Old conceptions die hard

Several participants were professionally active during the 1990s and early 2000s. While remembering guidelines recommending that the IUS only be prescribed to adult women who had given birth, they also recalled instances of exceptions. They said that exceptions were made when teenagers were deemed incapable of administering oral HCs, such as when adolescents had mental health issues, showed risky sexual behavior, or were under the care of child protective services. They also said it was done with teenagers who had gotten pregnant at a very young age and were admitted to hospital for an abortion. The IUS could then be inserted to prevent further pregnancies, sometimes without the consent of these young women. While all our participants said they perform their current professional tasks in line with updated guidelines, many suggested that attitudes generated from practices of the past are still alive today and serve as barriers against prescribing the IUS to adolescent women. They described misconceptions about the IUS, and they talked about misinformation on health being very difficult to disprove once disseminated in society:

*“And it’s so dangerous when something gets out and it’s wrong*,* because it is precisely like that; turning something around—that you can spend a lot of time doing”* (Public health nurse, age 43, 4 years of experience prescribing HCs to adolescents).

Two groups in particular were mentioned as sometimes carrying outdated advice. First, mothers of adolescents were referred to by several participants as being hesitant towards their daughters using the IUS:

*“And that thing about the IUS too*,* it’s just a myth*,* right? But that’s what was considered fact when they [the mothers] were young*,* so it’s no wonder they are saying this”* (Public health nurse, age 43, 4 years of experience prescribing HCs to adolescents).

Furthermore, situations where information from mothers conflicted with information from friends were described:

*“Some have heard from their moms that it [the IUS] is not something one can get as a young person*,* but then they have heard from their friends that they can get it*,* so they’re a bit curious about it*” (Midwife, age 43, 7 years of experience prescribing HCs to adolescents).

Upon being asked if mothers are skeptical only of the IUS, or also of other types of HCs, one participant said:

*“I think probably the IUS*,* since it’s a bit newer*,* and since that generation of moms was not used to having an IUS at a young age*,* so it’s probably*,* they might hear some stories from the moms that the IUS is not*…” (Public health nurse, age 39, 7 years of experience prescribing HCs to adolescents).

The second group described as sometimes being skeptical of prescribing the IUS to adolescent women were doctors, mainly family doctors:

*“And also the fact that other family doctors say that the IUS is not recommended for adolescents who have not given birth*,* I have heard that that idea is still out there. That they [the adolescents] have said that “my family doctor doesn’t recommend it because I am young and I have not given birth”. But this is not a required criterion for getting an IUS*,* so I think that the IUS—old myths are still alive*,* as it is only recently that adolescents can use it*,* or that it is recommended that they use it* …” (Public health nurse, age 39, 7 years of experience prescribing HCs to adolescents).

Some said that even the doctors working at the health centers could sometimes be restrictive, mainly with regard to sexual experience:

*“You could say*,* sometimes there are some that barely have had their sexual debut*,* and that one thinks about the IUS—in them one should not insert the IUS*,* or the doctors here with us say that they should be a bit more sexually active*,* well*,* they say*,* before possibly inserting one in them”* (Public health nurse, age 59, 13 years of experience prescribing HCs to adolescents).

Others pointed to similar assertions in the media:

*“…after all*,* there has been a lot of discussion about it in the media this summer. Among other things*,* there were quite a few doctors who stated that they [the teenagers] needed to be experienced with regard to sex*,* and that it could become a violation lying in the gynecological chair*,* and things like that”* (Public health nurse, age 52, 6 years of experience prescribing HCs to adolescents).

Another participant was a doctor herself, and she worked both at the health center for adolescents and as a family doctor at a family clinic. Her hesitation revolved around pain and discomfort:

*“And I feel like that setting can be a bit demanding*,* that is*,* inserting it in a successful manner. Making them relax and making sure they have an ok experience. One does not want them to have a very painful experience with something like that*,* that early*,* at such a young age*,* experiencing a lot of pain in the pelvic area. But*,* yes. But I will happily try if they want it*,* but I must admit that I am less…I am even more pro after they have given birth”* (General medical practitioner, age 44, 13 years of experience prescribing HCs to adolescents).

### Particular promotion of the intrauterine system to “vulnerable” adolescents

Most participants said that although the long-acting contraceptives—the IUS and the subdermal implant—are first-line recommendations for all adolescents when aiming to prevent pregnancy, healthcare professionals tend to promote these products more strongly to individuals they perceive as “vulnerable”. When asked what constitutes a vulnerable adolescent in their opinion, several examples were given. Most participants talked about being “disorganized” as a marker of being vulnerable, describing adolescents that for various reasons would have trouble remembering to take a pill every day. Moreover, lack of support at home, previous abortions, being cared for by child protective services, substance abuse, attention deficit hyperactivity disorder (ADHD) and other cognitive impairment and mental health issues were aspects put forth by many as contributing to someone being deemed vulnerable.

Most participants described making an extra effort to ensure these women got the IUS or the implant. One participant put it like this:

*“But then we also have certain vulnerable girls whom we think should get an implant or an IUS*,* and we put in some work with them*,* because they are very at-risk and vulnerable*,* perhaps also anxious*,* and not wanting to use anything at all. But many of them are not capable of administering oral contraceptive pills*,* so we have to put in some work with them*” (Public health nurse, age 57, 13 years of experience prescribing HCs to adolescents)*—*and her colleagues all agreed.

Another participant said about vulnerable adolescents that:

*“So those particular ones become really important for us to help in a good way*,* so they’re protected*,* so for them we go the extra mile. We notice that sometimes we really go the extra mile to get them in for an appointment for an IUS or an implant […] and so we keep at it until we have managed to help them”* (Public health nurse, age 49, 8 years of experience prescribing HCs to adolescents).

The IUS was also described by many as being recommended even more strongly than the implant, as it has certain advantages. Participants talked about it reducing bleeding, staying in place for several years, and having lower doses of progestin that mainly act locally in the uterus. As such, some said they might preferentially recommend the IUS for girls who have mental health issues:

*“Yes*,* this is the case if they are already struggling mentally. It depends a bit on what they are struggling with*,* but in most cases*,* yes”* (General medical practitioner, age 57, 22 years of experience prescribing HCs to adolescents).

Another participant mentioned mental health and substance abuse:

*“But then you have those who aren’t really able to—if I can put it that way—take care of themselves or remember to take a pill*,* and who might be dealing with mental health challenges or under some sort of mental health follow-up*,* maybe even substance abuse*,* where you’d typically recommend that they should get an implant*,* you know*,* or opt for an IUS”* (Public health nurse, age 56, 10 years of experience prescribing HCs to adolescents).

Her colleague agreed, but added:

“*Yes*,* but then I would actually refer them to their family doctor and have an IUS inserted*,* which has a much lower dosage and only acts locally—it only acts locally in the uterus*” (General medical practitioner, age 57, 22 years of experience prescribing HCs to adolescents), followed by the first colleague saying *“Yes*,* preferentially an IUS*,* yes”.*

Some also talked about certain adolescents exhibiting behaviors that made them vulnerable. They described these as being sexually active at a young age, sometimes interacting with older boys, being careless and not making good choices sexually:

“*They have difficulties setting boundaries and are easily taken advantage of by others—we can see they’re easily led by others”* (Public health nurse, age 59, 13 years of experience prescribing HCs to adolescents) and *“They’re simply a bit careless and maybe don’t fully understand the cues in sexual situations. As a result*,* they put themselves at greater risk in those situations—for abuse*,* for instance”* (Public health nurse, age 49, 8 years of experience prescribing HCs to adolescents).

Several participants also described intellectual abilities as playing a role:

*“We also have those who*,* well*,* intellectually don’t function at the same level as others their own age. Some have mild intellectual disabilities*,* and it’s important to protect them from becoming pregnant. In that respect*,* a long-acting contraceptive is a really good option”* (Public health nurse, age 60, 18 years of experience prescribing HCs to adolescents).

### What tips the scale: pros, peeves and other people

Despite sometimes guiding adolescents towards different HC products, all participants emphasized their freedom of choice. Aiming to inform their choices, adolescent women were described as navigating different sources of information and weighing perceived pros and cons. The IUS was described as coveted by many, and the most sought-after benefit in addition to preventing pregnancy was said to be reduced bleeding:

*“And about bleeding*,* that’s something teenagers care about. They don’t want to bleed*,* and they don’t want to bleed haphazardly. That is*,* they don’t want to be sitting at school and suddenly start bleeding. They want to control their bleeding”* (Public health nurse, 60, 18 years of experience prescribing HCs to adolescents).

Moreover, the fact that it is long-lasting and doesn’t require attention once inserted was described as valuable to teenagers:

“*Duration is important*,* yes. Because that’s something I notice*,* as I see the ones in upper secondary school. Some of them are like*,* well*,* they’re planning to go off to study and so on. And then I find that many of them tend to choose an IUS”* (Public health nurse, age 54, 13 years of experience prescribing HCs to adolescents).

The main reason for being skeptical towards the IUS was said to be related to the procedure of having an IUS inserted into one’s uterus. The healthcare professionals said adolescents typically fear pain and feel embarrassed about being exposed when in the gynecological chair:

*“Yes*,* you’re lying there in a gynecological exam chair*,* and everyone finds that a bit embarrassing*,* that it’s uncomfortable*,* and that it hurts. It does for everyone when the IUS is inserted”* (Public health nurse, age 60, 18 years of experience prescribing HCs to adolescents).

However, participants also reported that most adolescents go through with the procedure despite their fears. They described the health center setting as helpful when meeting frightened teenagers, as the health nurses and midwives working there spend as much time as necessary to calm them down, encourage, and comfort them:

*“But the advantage of working with adolescents in that group is that we’re really good at reassuring them*,* talking with them*,* joking around a bit*,* and we do have some time for that”* (Public health nurse, age 60, 13 years of experience prescribing HCs to adolescents).

Adolescents coming to the health center for contraceptive counselling were also said to be influenced by others’ opinions when choosing an HC. When asked who adolescent women mostly listened to, all participants agreed that friends were important. Sisters, mothers and aunts were described by many as conveyors of personal stories.

*One participant said: “They really focus on what their friends and siblings have said*,* you know*,* and they kind of fixate on that”* (Public health nurse, age 56, 13 years of experience prescribing) and another said: *“But if their mom has had a bad experience with something*,* that’s it—then they definitely don’t want whatever mom had*” (Public health nurse, age 57, 13 years of experience prescribing HCs to adolescents).

However, influencers and social media were also described as having become sources of information for many adolescent women during the last couple of years. Some said that this was relevant mostly for the oldest in this group:

*“TikTok brought a lot of confusion*,* or they got really scared of hormones*,* became skeptical”* (Public health nurse, age 56, 4 years of experience prescribing HCs to adolescents).

Despite the impact of friends, relatives and social media, most of the healthcare professionals said they felt that adolescent women were open to professional input. They said that even though they may have been told certain things prior to coming to the health center, they frequently changed their minds when given information based on research and medical guidelines:

*“They [the influencers] have a lot of power*,* to put it like that. Every time they [the adolescents] come in*,* I’m so grateful and happy*,* because then we can look into*,* well*,* what has been written*,* what has been said*,* what have you understood*,* what do you think about it—and then just break it all down with a bit of research and feedback to figure out what the actual facts are in all of this”* (Public health nurse, age 59, 13 years of experience prescribing HCs to adolescents).

## Discussion

The current study explored prescription of different HCs to adolescent users, with a particular interest in the intrauterine system. We find that although the IUS is now recommended for women of all reproductive ages, individual user characteristics may have an impact on who ends up choosing it or having it prescribed. Our analysis reveals that different considerations are weighed and balanced when choosing an HC, and that prescription of the IUS may still diverge from prescription of other HCs. The principal implication of these findings is that the subset of adolescents using the IUS may be subject to selection bias, thus limiting its representativeness of the broader adolescent population. Consequently, future quantitative studies risk confounding, unless potential selection biases are considered and controlled for.

### Conceivable selection biases in the population of IUS users

While accounting for all possible factors contributing to bias may be implausible, several conceivable selection biases were identified in this study. First, the reluctance toward adolescent IUS use described in our material may introduce bias through an indirect, gatekeeper-type manner. If mothers or family doctors who are otherwise reluctant make exceptions only when there is a specific therapeutic need and, in doing so, affect which HC is prescribed, the resulting population of IUS users might not be representative of the general female adolescent population. For instance, therapeutic indications for prescribing the IUS to teenagers include medical conditions such as endometriosis, menorrhagia, dysmenorrhea, polycystic ovary syndrome and obesity [reviewed in 8]. These are conditions that are also associated with increased levels of psychological distress, depression, and/or anxiety [27–30]. If young women suffering from these conditions tend to have the IUS prescribed more often than women who do not, then these individuals could bias the IUS-using population in a direction of higher average scores on measures of depressive symptoms.

Second, while our participants describe health center prescription practices that promote equal access to each product for all, they also outline a set of user characteristics that elicit increased recommendations for choosing the IUS, along with additional efforts in facilitating an appointment to have it inserted. These characteristics are summarized as making the individual vulnerable, and one of the main examples given is “being disorganized” and unable to remember taking a pill every day. Being disorganized could simply be part of normal teenage life, or it could represent an innocuous personality trait—but it could also reflect varying degrees of executive dysfunction. Disrupted executive function is associated with both early and later stages of several psychiatric disorders that involve mood disturbances, such as depression and anxiety [31], bipolar disorder [32] and ADHD [33, 34]. If this is the case for some of these girls, this too has the potential for biasing the IUS-using population in a direction where mental health issues represent pre-existing conditions that serve as indications for having the IUS prescribed, rather than being a consequence of using the IUS. Similarly, the cited substance abuse, child protection service involvement, decreased intellectual abilities and personality traits leading to risky sexual behaviors could also represent risk factors for poor mental health outcomes [35–38]. In addition to these potential or indirect links to mental health issues, pre-existing mental health conditions were also referred to directly by our informants, as a reason to promote the IUS more strongly than other HC products. While these assessments make considerable clinical sense, from a research perspective it is important to note that they can confound IUS studies unless controlled for.

The third aspect we considered relevant for HC prescription, adolescents’ process of choosing, could also have implications for who ends up constituting the IUS-using population. Our participants described fear or embarrassment when having the IUS inserted, as a factor in most teenagers’ choice. The ability to overcome these hurdles could determine whether they ask for a prescription for the IUS or not. Increased levels of the personality trait neuroticism could prevent an individual from going through with the procedure, and as such, the IUS-using population could consist of fewer individuals with neurotic propensities. If this is the case, the IUS-using population could be biased in the opposite direction of what is described above, as lower levels of neuroticism are associated with lowered risk of depression [39]. However, all participants said that most adolescents coming to the health center manage to have the procedure done, despite being scared or anxious up front, as the benefits associated with the IUS usually counteract the initial fear. Nevertheless, the most anxious individuals may not even consider an IUS, and as such, they may never encounter healthcare professionals in this regard. Thus, while not a prominent feature in our material, neurotic traits cannot be ruled out as affecting who chooses to use the IUS and who chooses not to. Additional influences, such as the opinions of friends, female relatives, and influencers, could further sway adolescents’ decisions in one direction or another. Both friends and relatives, however, were described by our informants as heterogeneous groups in terms of advice. Aside from some of the mothers being skeptical towards the IUS, we could not detect any clear pattern within these groups, neither in terms of specific products, nor in terms of using or not using HCs. Influencers on social media, however, were described by most participants as discouraging the use of any HC. Their promoting “being natural” could lead to fewer adolescents using HCs, but influencers were not said to denigrate specific types of HCs more than other types. Therefore, it seems unlikely that they currently affect the population using the IUS when comparing with users of other products. However, these impacts could be subject to rapidly shifting trends, and they might not be the same in different countries or regions. Moreover, if certain types of young women are more impressionable, and more easily swayed by influencers, the population using any HC could end up being different from the population not using hormonal products at all.

### Implications for future quantitative research

As data for the current study were collected and analyzed qualitatively, we cannot determine to what degree our results can be replicated in other samples or settings. However, the study has identified possible background variables that, unless considered and accounted for, may distort interpretations of research findings. Moreover, if cases that are rare in the general population are overrepresented in a selected sample, the resulting conclusions may be flawed. The extent of confounding phenomena present in the current IUS-using population cannot be determined within this study, but our findings point to possible confounders that warrant further investigation. Although circumstances have changed since the 1990s and early 2000s, and intrauterine systems are now widely used among adolescents, the factors identified in this study could still influence patterns of IUS use. Consequently, these factors should be assessed in future quantitative studies aiming to disentangle cause and effect as related to IUS use and mental health.

### Limitations

The transferability of our findings should be considered in light of the specific healthcare context in which the study was conducted. Norway’s universal healthcare system, the established network of publicly funded health centers for adolescents and the prominent role of nurses and midwives in contraceptive counseling represent structural conditions that may not be present in other settings. In healthcare systems where contraceptive services are delivered differently, through primary care physicians, private providers, or with greater resource constraints, the factors influencing prescription practices may differ. Readers are encouraged to consider the fit between these contextual features and their own setting when assessing the relevance of the findings. However, changes in recommendations for prescribing the IUS to adolescent women have happened on a global scale, and there is reason to believe that the topic and our findings may be relevant also elsewhere. Furthermore, while health centers in Norway have an overwhelmingly high prevalence of female employees (99.7% of both public health nurses and midwives in Norway are female) [40, 41], the fact that our sample consisted entirely of female informants should also be mentioned as a potential limitation. Moreover, while conducting interviews in group settings may provide dynamic and synergistic discussions, there is also a possibility that more controversial thoughts or opinions may be stifled in the presence of others. Further, self-reporting may in itself be associated with an inherent bias, regardless of design, and self-reported practices may differ from actual clinical behavior. Additionally, two of our groups only had two participants each (the rest consisted of four, four, five and six, respectively), and this should be mentioned as a limitation. We decided to allow for the smaller groups, in order to include also smaller health stations based in rural or suburban areas. Finally, in the current study we only talked to prescribers of HCs, and not to adolescents themselves. This could represent a limitation, as additional insight on user preference may have been gained by talking to adolescents. However, we chose this approach as we considered healthcare professionals to have a better overview of the variety of factors that could affect prescription than the teenagers themselves. Unlike individual adolescents, who can only speak to their own experiences and choices, healthcare professionals observe patterns across many consultations and diverse patient groups, positioning them to offer insights into what systematically influences prescribing practices rather than what influences any single encounter.

## Conclusions

Adolescent hormonal contraceptive use has changed during the past couple of decades. Whereas intrauterine systems previously were reserved for adult women who had given birth, these products are now both used by and recommended for adolescents. However, our analysis shows that several factors related to user characteristics and conceptions of the past could still influence who chooses the IUS, or has it prescribed. These characteristics should be assessed in future quantitative studies, to avoid confounding when drawing causal inferences concerning adolescent IUS use and mental health.

## Supplementary Information

Supplementary Material 1.

Supplementary Material 2.

## Data Availability

Given the qualitative nature of the study, interview transcripts are not publicly available due to the risk of participant identification. However, these may be obtained from the corresponding author upon reasonable request.

## Abbreviations

HC: Hormonal contraceptives
IUS: Intrauterine system
ADHD: Attention deficit/hyperactivity disorder
