# Supplementary material for: Particular promotion of the intrauterine system to “vulnerable” adolescents: a qualitative exploration of hormonal contraceptive prescription practices
Source: Reprod Health. 2026 Jun 17;23:162. doi: 10.1186/s12978-026-02388-9 (PMC13551700; doi:10.1186/s12978-026-02388-9)
Supplement: Supplementary file 1 [file 12978_2026_2388_MOESM1_ESM.pdf]

Interview guide

Thank you for agreeing to take part in this study. If anyone has questions before we start the interview, please let us know.

Today we want to explore various issues related to prescribing hormonal contraception to teenagers and young women, and we want to hear about your experiences from your everyday clinical practice.

Several hormonal contraceptive products are currently available on the Norwegian market. These differ in terms of hormone content and dosage, as well as regimen and route of administration. Today we want to talk about the intrauterine system, the subdermal contraceptive implant, and oral contraceptive pills.

1) Choice of method

First, we would like to talk a bit about choosing and recommending different methods.

- When young women come to you and they want hormonal contraception, what do they cite as reasons for wanting contraception?
- When young women are choosing between different methods, who do they get advice from? Who do they listen to?
- When these women come to you, what do you as health professionals base your recommendation of a method on?
  - Which guidelines? Who do you get advice from? Who do you listen to?
  - Do you have different recommendations for the youngest compared with those who are 21 or older?
    - If so, where would you draw the line?
  - Do different individuals receive different recommendations?
    - If so, who, and in what way?
  - Do you distinguish between different types of oral contraceptive pills?
    - If so, what is recommended for whom?

2) The intrauterine system and the subdermal contraceptive implant

Now we would like to talk more specifically about the intrauterine system and the contraceptive implant, compared with oral contraceptive pills:

- When young women consider getting an intrauterine system inserted, what is important to them?
  - Are there barriers to getting an intrauterine system?
    - If so, what are these?
  - Are there benefits that they seek?
    - If so, what are these?

    - Are there differences between the very youngest and the somewhat older adolescents?
- What about the contraceptive implant—what is important to young women here?
    - Are there barriers to getting an implant?
        - If so, what are these?
    - Are there benefits that they seek?
        - If so, what are these?
    - Are there differences between the very youngest and the somewhat older adolescents?
- If an intrauterine system is being considered, which factors related to the adolescents are important for you as health professionals to take into account?
    - Are there reasons to recommend an intrauterine system rather than oral contraceptive pills, or oral contraceptive pills over the intrauterine system?
    - Are there reasons not to recommend an intrauterine system?
    - Do you take other factors into account for the youngest compared with those who are slightly older?
    - Do you take other considerations into account when prescribing an intrauterine system to this age group than to women over 21?
- What about the contraceptive implant—what factors related to the adolescents do you as health professionals take into account?
    - Are there reasons to recommend an implant rather than oral contraceptive pills, or oral contraceptive pills over an implant?
    - Are there reasons not to recommend an implant?
    - Do you take other factors into account for the youngest compared with those who are slightly older?
    - Do you take other considerations into account when prescribing an implant to this age group than to women over 21?

3) Side effects

Now we would like to ask about side effects and hormonal contraception.

- Which side effects do young women who come to you report?
    - With the use of different types of oral contraceptive pills?
    - With the use of an intrauterine system?
    - With the use of an implant?
- What recommendations do you as health professionals give to young women who report side effects from a specific method?
    - How long do you recommend that they stay on that method before potentially switching?

4) Changes in intrauterine system prescription guidelines and practices

Finally, guidelines for prescribing intrauterine systems have changed since the 1990s and early 2000, when this contraceptive method was recommended mainly for adult women who had given birth.

- For those of you who were professionally active during that time, do you remember if the intrauterine system was described to teenagers, although this was counter to medical guidelines of that time?
    - If so, under what circumstances?

Before we say thank you for today, one final, more open question: Do one or more of you have anything to add? Anything that should have been commented on?
