# Supplementary material for: Particular promotion of the intrauterine system to “vulnerable” adolescents: a qualitative exploration of hormonal contraceptive prescription practices
Source: Reprod Health. 2026 Jun 17;23:162. doi: 10.1186/s12978-026-02388-9 (PMC13551700; doi:10.1186/s12978-026-02388-9)
Supplement: Supplementary file 2 [file 12978_2026_2388_MOESM2_ESM.pdf]

COREQ (COnsolidated criteria for REporting Qualitative research) Checklist

A checklist of items that should be included in reports of qualitative research. You must report the page number in your manuscript where you consider each of the items listed in this checklist. If you have not included this information, either revise your manuscript accordingly before submitting or note N/A.

Topic	Item No.	Guide Questions/Description	Reported on Page No.
Domain 1: Research team and reflexivity			
Personal characteristics			
Interviewer/facilitator	1	*Which author/s conducted the interview or focus group?*<br>Marita Kallesten Brønnick (MKB) and Tore Tjora (TT)	10
Credentials	2	*What were the researcher's credentials? E.g. PhD, MD*<br>MSc, PhD Candidate (MKB) and PhD (TT, Jone Ravndal Bjørnestad (JRB) and Daniel Adrian Lungu (DAL))	10
Occupation	3	*What was their occupation at the time of the study?*<br>PhD Candidate (MKB) and Associate Professor (TT and DAL), Full Professor (JRB)	10
Gender	4	*Was the researcher male or female?*<br>Female (MKB) and male (TT, JRB and DAL)	10
Experience and training	5	*What experience or training did the researcher have?*<br>Nursing, master in Sexology, master in Applied neuroscience (MKB), Clinical Psychology (TT and JRB), and Health Services Research (DAL)	10
Relationship with participants			
Relationship established	6	*Was a relationship established prior to study commencement?*<br>No	N/A
Participant knowledge of the interviewer	7	*What did the participants know about the researcher? e.g. personal goals, reasons for doing the research*<br>Name, occupation, university affiliation	10
Interviewer characteristics	8	*What characteristics were reported about the interviewer/facilitator? e.g. Bias, assumptions, reasons and interests in the research topic*<br>Interest in the research topic as part of a PhD project	10
Domain 2: Study design			
Theoretical framework			
Methodological orientation and Theory	9	*What methodological orientation was stated to underpin the study? e.g. grounded theory, discourse analysis, ethnography, phenomenology, content analysis*<br>Critical realism, thematic analysis	8
Participant selection			
Sampling	10	*How were participants selected? e.g. purposive, convenience, consecutive, snowball*<br>Purposive	9

Topic	Item No.	Guide Questions/Description	Reported on Page No.
Method of approach	11	*How were participants approached? e.g. face-to-face, telephone, mail, email*<br>Phone and e-mail via head of department/-center	9
Sample size	12	*How many participants were in the study?*<br>23	9
Non-participation	13	*How many people refused to participate or dropped out? Reasons?*<br>Two centers, illness among staff	9
Setting			
Setting of data collection	14	*Where was the data collected? e.g. home, clinic, workplace*<br>Workplace (Health centers for adolescents)	9
Presence of non-participants	15	*Was anyone else present besides the participants and researchers?*<br>No	9
Description of sample	16	*What are the important characteristics of the sample? e.g. demographic data, date*<br>The sample consisted of female employees of health centers for adolescents in the southwestern part of Norway. Nineteen were public health nurses, two were midwives, and two were general medical practitioners. Age range was 38-63, mean age was 52, and years of experience prescribing HCs to young women ranged from 1.5 to 22, with a mean of 10.	9
Data collection			
Interview guide	17	*Were questions, prompts, guides provided by the authors? Was it pilot tested?*<br>Yes	10
Repeat interviews	18	*Were repeat interviews carried out? If yes, how many?*<br>No	N/A
Audio/visual recording	19	*Did the research use audio or visual recording to collect the data?*<br>Yes, audio	11
Field notes	20	*Were field notes made during and/or after the interview or focus group?*<br>No	N/A
Duration	21	*What was the duration of the interviews or focus group?*<br>90 minutes	10
Data saturation	22	*Was data saturation discussed?*<br>Yes, we use the “information power” framework	11
Transcripts returned	23	*Were transcripts returned to participants for comment and/or correction?*<br>No	N/A

Topic	Item No.	Guide Questions/Description	Reported on Page No.
Domain 3: Analysis and findings			
Data analysis			
Number of data coders	24	*How many data coders coded the data?* Two	12
Description of the coding tree	25	*Did authors provide a description of the coding tree?* No, we generated an initial coding map, that was subsequently shared with the full team and condensed	12
Derivation of themes	26	*Were themes identified in advance or derived from the data?* Derived from data	12
Software	27	*What software, if applicable, was used to manage the data?* NVivo	12
Participant checking	28	*Did participants provide feedback on the findings?* No	N/A
Reporting			
Quotations presented	29	*Were participant quotations presented to illustrate the themes/findings? Was each quotation identified? e.g. participant number* Yes, participant age, background and years of experience were reported for each quote	13-23
Data and findings consistent	30	*Was there consistency between the data presented and the findings?* Yes	12-27
Clarity of major themes	31	*Were major themes clearly presented in the findings?* Yes	12-23
Clarity of minor themes	32	*Is there a description of diverse cases or discussion of minor themes?* No	N/A

Developed from: Tong A, Sainsbury P, Craig J. Consolidated criteria for reporting qualitative research (COREQ): a 32-item checklist for interviews and focus groups. *International Journal for Quality in Health Care.* 2007. Volume 19, Number 6: pp. 349–357

Once you have completed this checklist, please save a copy and upload it as part of your submission. DO NOT include this checklist as part of the main manuscript document. It must be uploaded as a separate file.
